# Unklare bilaterale perilimbale Schwellung

**DOI:** 10.1007/s00347-020-01046-0

**Published:** 2020-02-03

**Authors:** H. Heinen, M. Notara, N. Loreck, R. S. Grajewski, C. Cursiefen

**Affiliations:** Zentrum für Augenheilkunde, Uniklinik Köln, Kerpener Str. 62, 50937 Köln, Deutschland

Eine 42-jährige Patientin stellte sich erstmalig in unserem Hause vor, da sie seit etwa 1 Jahr einen „Ring um die Pupille“ bemerkt habe. Sie beklagte weiterhin eine häufige Rötung der Augen und Trockenheitsgefühle. Die Patientin gab an, seit dem 12. Lebensjahr konstant weiche Kontaktlinsen (KL) zum Teil als Monats- oder Jahreslinsen zu tragen.

In der Spaltlampenmikroskopie zeigte sich der in Abb. 1 abgebildete Befund. Der Visus betrug rechts ccs sph −7,75 zyl −2,5 × 163 = 1,0 und links bei Schielamblyopie ccs sph −7,5 zyl −1,5 × 3 = 0,25, der intraokulare Druck (IOD) (Icare® TA01i, Icare Finland Oy, Vantaa, Finnland) beidseits 12 mm Hg. Die Hornhaut zeigte sich in der Pachymetrie zentral 467/497 µm dick.

**Figure 1:**
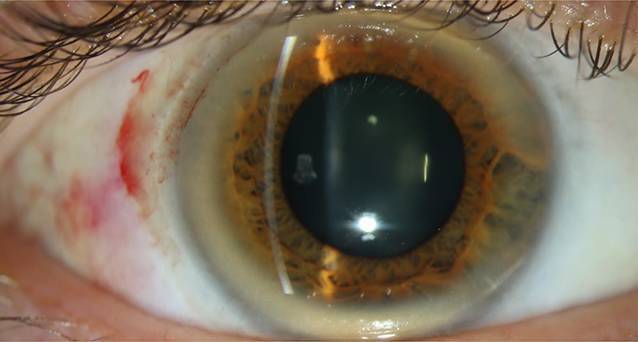

Wir indizierten zunächst eine Therapie mit befeuchtenden Augentropfen (AT), Lidrandpflege und empfahlen der Patientin, keine KL mehr zu tragen.

Verlaufskontrollen bei uns erfolgten alle 3 bis 6 Monate. Die Patientin versicherte, eine strenge KL-Karenz einzuhalten. Im Verlauf kam es insbesondere am rechten Auge trotzdem zu einem Fortschreiten des Befundes. Etwa 1 Jahr nach Erstvorstellung sahen wir den in Abb. 2 abgebildeten Befund.

**Figure 2:**
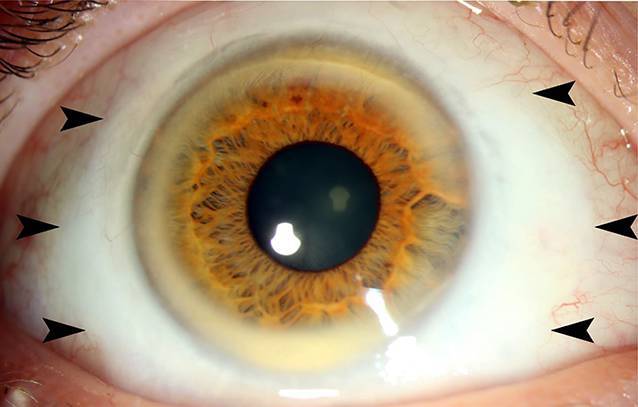

Anamnestisch ergab sich kein Hinweis auf eine stattgefundene Skleritis. Eine kardiovaskuläre Abklärung ergab keinen Hinweis auf eine Dyslipidämie oder Karotisstenose. Ein Uveitissuchprogramm zeigte sich unauffällig: Antineutrophile zytoplasmatische Antikörper (ANCAs), Rheumafaktor (RF), Angiotensin-Converting-Enzyme (ACE) waren negativ; antinukleäre Antikörper (ANAs) waren grenzwertig erhöht ohne spezifisches Antigen und somit ohne diagnostische Relevanz. Es ergab sich kein Hinweis auf eine systemische Infektion oder Systemerkrankung. Bei Photophobie, Epiphora und Schmerzen indizierten wir eine antientzündliche Therapie mit Ciclosporin A AT (Augentropfen) (1 mg/ml) z. N. (zur Nacht).

Zwei Monate später stellte sich die Patientin erneut vor, nachdem ihr vor 3 Wochen ein stumpfer Gegenstand in das rechte Auge gekommen sei. Sie beklagte eine Visusminderung und Metamorphopsien. Eine Therapie mit Prednisolonacetat AT (1 %) 6‑mal täglich über 1 Woche habe nicht geholfen.

Spaltlampenmikroskopisch zeigte sich der Befund aus Abb. 3a. Der Seidel-Test war negativ. Es zeigten sich eine Papillenschwellung und Aderhautfalten e vacuo (Abb. 3c, d). Wir sahen keine entzündliche Aktivität. Der Visus betrug ccs 0,6. Der IOD betrug appl. 4 mm Hg. Anamnestisch sei dieser seit dem Trauma so niedrig gewesen.

**Figure 3:**
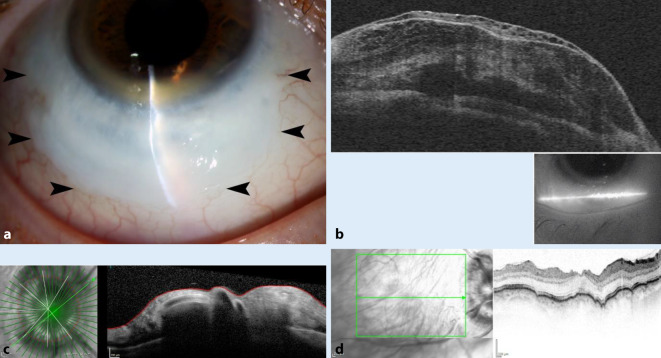

## Wie lautet Ihre Diagnose?

Aufgrund der fehlenden Zeichen für eine entzündliche Genese und unauffälliger Autoimmundiagnostik diagnostizierten wir ein posttraumatisches Filterkissen im Sinne einer gedeckten Perforation bei 4 bis 8 Uhr mit Bulbushypotonie, Stauungspapille und Makulafalten e vacuo im Rahmen eines massiven Kontaktlinsentrageschadens mit chronisch irritativer Entzündung und Hypoxieschaden sowie partieller Limbusstammzellinsuffizienz. Eine marginale Degeneration Terrien wurde nicht ganz ausgeschlossen. Eine Beteiligung einer marginalen Degeneration Terrien schlossen wir nicht aus.

Die Patientin wurde zunächst mit einer 16 mm KL, einer Schutzklappe und Gentamicin (5 mg/ml)/Dexamethason (1 mg/ml)-Verband versorgt. Am folgenden Tag zeigten sich eine Irisinkarzeration sowie limbusnah eine Skleranekrose mit weiter bestehender Papillenschwellung und intraokulärer Hypotonie, sodass wir uns dazu entschieden, die Patientin zu operieren. Intraoperativ zeigte sich limbusnah von 4 bis 8 Uhr eine nekrotische Bindehaut. Diese wurde abgetragen. Darunter zeigte sich ein interkalares Staphylom mit Iris- und Uveaprolaps. Wir führten eine Deckung mittels semilunarer anteriorer lamellärer Keratoplastik (DALK) à chaud und ausgedünntem Sklerapatch durch. Ein gekürztes OP-Video findet sich im MediaContainer.

**Figure 4:**
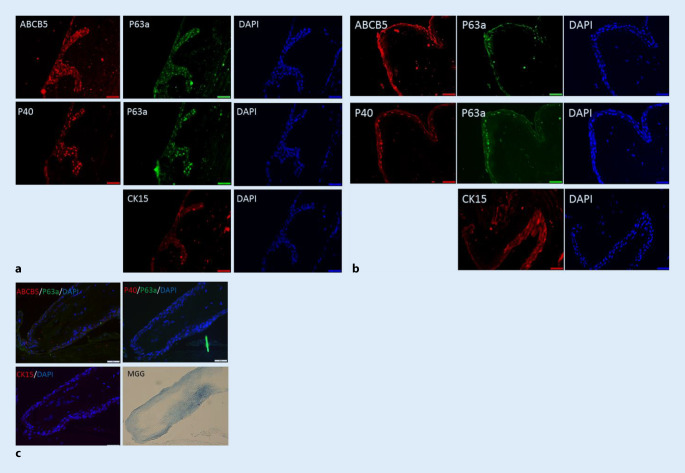

Die histopathologische Untersuchung ergab eine subepitheliale plaqueartige hyaline Fibrose bei länger bestehender chronischer irritativer Entzündung ohne Hinweis auf eine spezifische Entzündung, Elastose oder Malignität. Kulturell ergab sich kein Nachweis von Bakterien oder Pilzen. Immunhistochemische Färbungen zeigten in einigen Bereichen eine deutliche Abnahme der limbalen Stammzellen, während sie in anderen nur leicht reduziert waren (Abb. 4).

**Figure 5:**
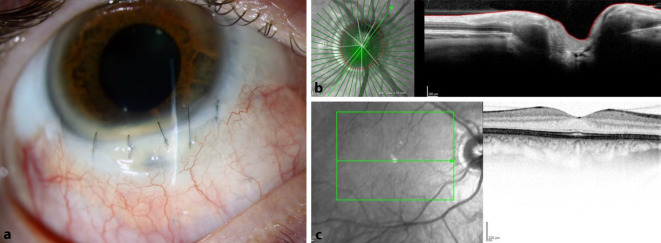

Wir entließen die Patientin am 5. postoperativen Tag mit gut verheiltem Defektareal, einem IOD von 10 mm Hg und einem Visus (ccs) von 0,1 am rechten Auge. Die indizierte Therapie bestand aus Dexamethason (1 mg/ml)/Gentamicin (5 mg/ml) AT 4‑mal täglich, einer Dexamethason (0,3 mg/1 g)/Gentamicin (1 mg/g) AS z. N. und Pilocarpin 2 % (20 mg/1 g) AT 3‑mal täglich. Dies wurde im Verlauf reduziert. Der postoperative Heilungsverlauf zeigte sich darunter insgesamt stabil mit klarer Hornhaut und adaptierter Bindehaut. Aderhautfalten und Papillenschwellung waren komplett rückläufig (Abb. 5). Der Visus betrug 9 Wochen nach der Operation ccs −8 sph −4,75 × 65 zyl = 1,0.

## Diskussion

Wir sahen bei der Patientin ein perilimbales Ödem mit limbusnaher Bindehautischämie und Skleranekrosen ohne sichtbare Entzündungsaktivität. Abgelaufene Skleritiden waren anamnestisch nicht zu eruieren. Es ergab sich kein Hinweis auf eine kardiovaskuläre Genese mit chronischer Limbusischämie oder auf eine Vaskulitis-assoziierte periphere ulzerative Keratitis. Auch histopathologisch ergab sich kein Hinweis auf eine vaskulitische, dysplastische oder granulomatöse Genese. Der klinische Befund zeigte sich nicht typisch für einen Ulcus Mooren oder eine pelluzide marginale Hornhautdegeneration. Aufgrund der Lipidablagerungen und peripheren Stromaverdünnungen schlossen wir jedoch eine Beteiligung eines Morbus Terrien nicht aus. Zwar zeigten sich Bereiche mit einer verminderten Anzahl von limbalen Stammzellen, das Epithel war jedoch gänzlich intakt, und die Neovaskularisationen waren begrenzt. Sekundär kam es durch ein Trauma zur Ruptur der Sklera mit einem konsekutiven Filterkissen und okulärer Hypotension und Stauungspapille e vacuo.

Wir gingen von einem Schaden durch Kontaktlinsenabusus aus. Eine ständige mechanische Irritation durch die KL, eine induzierte Tränenfilminstabilität, Hypoxien und druckbedingte vaskuläre Ischämien unterhielten chronische subklinische Entzündungsreaktionen mit Schädigung und Nekrose der subepithelialen Schichten [1, 2]. Die jahrelange exzessive Anwendung von Kontaktlinsen als Monats- und Jahreslinsen haben hier in einer individuellen Prädisposition ebenfalls zu einer Limbusschädigung geführt. Interessanterweise zeigten sich die verbliebenen Stammzellen erstaunlich widerstandsfähig und konnten das Epithel erhalten. Aufgrund der ausgedünnten und nekrotischen subepithelialen Bereiche führte jedoch schon ein leichtes Trauma zu einer Perforation der Sklera.

**Diagnose:** Gedeckte Perforation durch chronischen Kontaktlinsenschaden

Die Limbusstammzellinsuffizienz ist gekennzeichnet durch Dysfunktion oder Verlust der limbalen Stammzellen der Kornea. Diese besitzen die Fähigkeit zur Ausdifferenzierung und Regeneration des Hornhautepithels und bilden eine Barriere gegenüber der Konjunktiva. Nach ihrem Verlust kommt es zu einem Einwachsen konjunktivaler Zellen auf die Hornhaut [3]. Bei 5 % aller Träger finden sich klinische Anzeichen. Prädisponierend sind v. a. weiche KL sowie die Tragedauer [4, 5].

## Fazit für die Praxis

- Bei 5 % aller Kontaktlinsen(KL)-Träger finden sich Anzeichen einer Limbusstammzellinsuffizienz.
- Leichte Formen können konservativ mit einer KL-Karenz und einer Therapie des trockenen Auges behandelt werden.
- Schwere und schwerste Formen des Kontaktlinsentrageschadens können zu hypoxisch-ischämischen Schäden und zur Limbusstammzellinsuffizienz führen.
- Im Rahmen eines Traumas kann es bei ausgedünnter Sklera zur Ruptur kommen.
- Die limbalen Stammzellen zeigten sich hier erstaunlich widerstandsfähig.

## Caption Electronic Supplementary Material


